# Gesundheitsrisiken durch die Bewässerung von Nutzpflanzen mit aufbereitetem Abwasser, das Antibiotikarückstände, Resistenzgene und resistente Mikroorganismen enthält

**DOI:** 10.1007/s00103-023-03710-7

**Published:** 2023-05-26

**Authors:** Kornelia Smalla, Jan Kabisch, Gregor Fiedler, Jens Andre Hammerl, Bernd-Alois Tenhagen

**Affiliations:** 1 Institut für Epidemiologie und Pathogendiagnostik, Julius Kühn-Institut (JKI), Braunschweig, Deutschland; 2 Institut für Mikrobiologie und Biotechnologie, Max Rubner-Institut (MRI), Kiel, Deutschland; 3 Abteilung Biologische Sicherheit, Bundesinstitut für Risikobewertung (BfR), Max-Dohrn-Str. 8–10, 10589 Berlin, Deutschland

**Keywords:** Aufbereitete Abwässer, Antibiotikaresistenz, Arzneimittelrückstände, Pflanzenverfügbarkeit, Lebensmittelsicherheit, Water reuse, Antimicrobial resistance, Drug residues, Bioavailability, Food safety

## Abstract

Diese Übersichtsarbeit beschreibt Effekte und mögliche Gesundheitsrisiken durch resistente Mikroorganismen, Resistenzgene und Biozid- und Arzneimittelrückstände, die durch die Nutzung von aufbereitetem Abwasser zur Bewässerung von Nutzpflanzen entstehen können. Dabei fokussiert die Arbeit auf spezifische Aspekte dieser Kontaminanten und ihrer Interaktionen, strebt jedoch keine allgemeine Bewertung der mikrobiologischen Belastungen an.

In aufbereitetem Abwasser werden regelmäßig Rückstände von antibiotisch wirksamen Arzneimitteln, resistente Mikroorganismen und Resistenzgene nachgewiesen. Diese beeinflussen das Boden- und Pflanzenmikrobiom und können von den Pflanzen aufgenommen werden. Mit einer Interaktion von Antibiotikarückständen und Mikroorganismen ist vor allem vor der Ausbringung der aufbereiteten Abwässer zu rechnen. Sie kann allerdings auch das Pflanzenmikrobiom beeinflussen, inklusive der Gesamtheit seiner Resistenzgene (Resistom). Eine besondere Problematik ergibt sich dadurch, dass Pflanzen als Lebensmittel häufig roh, also ohne keimreduzierende Verarbeitungsschritte verzehrt werden. Gründliches Waschen pflanzlicher Lebensmittel beeinflusst das Pflanzenmikrobiom nur geringfügig. Zerkleinerungsschritte bei der Verarbeitung können hingegen zur Vermehrung vorhandener Mikroorganismen beitragen, so dass danach eine gründliche Kühlung erforderlich ist.

Bei der Aufbereitung von Abwässern, die zur Bewässerung von Nutzpflanzen eingesetzt werden sollen, werden neue Verfahren zur Elimination von Mikroschadstoffen und Mikroorganismen benötigt, um das Risiko einer verstärkten Exposition von Verbraucherinnen und Verbrauchern gegenüber übertragbaren Resistenzgenen und resistenten Bakterien weiter zu reduzieren.

## Abstract (en)

This review describes the effects and potential health risks of resistant microorganisms, resistance genes, and residues of drugs and biocides that occur when re-using wastewater for crop irrigation. It focusses on specific aspects of these contaminants and their interactions, but does not provide a general risk assessment of the microbial load when using reclaimed water.

Antimicrobial residues, antimicrobial resistant microorganisms, and resistance genes are frequently detected in treated wastewater. They have effects on the soil and plant-associated microbiota (total associated microorganisms) and can be taken up by plants. An interaction of residues with microorganisms is mainly expected before using the water for irrigation. However, it may also occur as a combined effect on the plant microbiome and all the abundant resistance genes (resistome). Special concerns are raised as plants are frequently consumed raw, that is, without processing that might reduce the bacterial load. Washing fruits and vegetables only has minor effects on the plant microbiome. On the other hand, cutting and other processes may support growth of microorganisms. Therefore, after such process steps, cooling of the foods is required.

Further progress has to be made in the treatment of wastewater that will be used for crop irrigation with respect to removing micropollutants and microorganisms to minimize the risk of an increased exposure of consumers to transferable resistance genes and resistant bacteria.

## Einleitung

Die Nutzung aufbereiteten Abwassers zu Bewässerungszwecken im Pflanzenbau ist bereits in vielen Regionen der Welt notwendig. Sie wird im Hinblick auf zunehmende Trockenperioden auch für die Landwirtschaft in Europa diskutiert. Da die Verordnung (EU) 2020/741 des Europäischen Parlaments und des Rates eine solche Bewässerung mit Einschränkungen vorsieht, soll dieser Übersichtsartikel den derzeitigen wissenschaftlichen Kenntnisstand und die sich daraus ergebende Risikobewertung für Deutschland darstellen [1]. Es wird diskutiert, ob für Rückstände von Antibiotika‑, Metall- und Biozidverbindungen bzw. das Vorhandensein von resistenten Bakterien besondere Vorsichtsmaßnahmen erforderlich sind, die über die im Hinblick auf pathogene Mikroorganismen ohnehin erforderlichen Maßnahmen hinausgehen.

Bewässerungswasser gilt als wichtige Quelle für den Eintrag von Antibiotika‑, Metall- und Biozidverunreinigungen sowie von antibiotikaresistenten Bakterien in Nutzpflanzenkulturen [2]. Es wird ein direkter Zusammenhang zwischen der Wasserqualität und der Kontamination von roh verzehrtem Obst und Gemüse vermutet. Das Vorkommen von Mikroschadstoffen, wie Antibiotikarückständen, und deren Aufnahme in die Pflanze kann sowohl die Zusammensetzung des natürlichen Pflanzenmikrobioms beeinflussen als auch die Etablierung der mit dem aufbereiteten Abwasser eingebrachten Keime. Das Pflanzenmikrobiom wiederum kann einen direkten Einfluss auf das menschliche Darmmikrobiom nehmen [3].

Zum Schutz vor lebensmittelbedingten Erkrankungen durch pathogene Bakterien, Viren und Parasiten durch Rohverzehr von frischem Obst und Gemüse sind entsprechende Anforderungen an die Wasserqualität zu berücksichtigen [4]. Mit diesem Übersichtsartikel wird gezeigt, dass die Qualität des zur Bewässerung genutzten aufbereiteten Abwassers auch im Hinblick auf die Antibiotikaresistenz-Problematik von großer Bedeutung ist.

Einerseits enthält aufbereitetes Abwasser Nährstoffe für das Pflanzenwachstum, andererseits sollten Mikroschadstoffe, die antibiotikaresistente Bakterien selektieren und die Übertragung von Genen zwischen Bakterien (horizontaler Gentransfer) stimulieren, vor der Nutzung von aufbereitetem Abwasser aus dem Bewässerungswasser entfernt werden [5].

Aufbereitete Abwässer enthalten deutlich geringere Rückstandskonzentrationen wie Antibiotika‑, Metall- und Biozidverbindungen als z. B. Gülle. Allerdings übersteigt bei einer regelmäßigen Bewässerung die Gesamtmenge der aufbereiteten Abwässer die der ausgebrachten Gülle deutlich, so dass es über die Zeit zu einer stärkeren Exposition des Bodens und der Pflanzen gegenüber diesen Substanzen kommen kann.

Wesentlich für die Fragestellung dieses Artikels sind einerseits der Gehalt an antimikrobiell wirksamen Substanzen im aufbereiteten Abwasser sowie der Gehalt an resistenten Mikroorganismen und ggf. freier DNA, die Resistenzgene tragen kann. Zweitens ist zu prüfen, in welchem Umfang sowohl die resistenten Mikroorganismen als auch die Rückstände von Arzneimitteln von den Pflanzen aufgenommen werden bzw. auf den Pflanzen verbleiben. Drittens ist zu fragen, in welchem Umfang übliche Maßnahmen, z. B. das Waschen von pflanzlichen Lebensmitteln vor dem Verzehr, zu einem relevanten Rückgang solcher Mikroorganismen und ggf. der Rückstände führen, der über den Effekt der Entfernung von Bodenpartikeln hinausgeht.

Dieser Übersichtsartikel strebt keine umfassende Risikobewertung der möglichen Exposition von Verbraucherinnen und Verbrauchern gegenüber resistenten Mikroorganismen, Resistenzgenen und Rückständen von Arzneimitteln durch die Bewässerung von Nutzpflanzen mit aufbereitetem Abwasser an, sondern fokussiert auf mögliche Aspekte, die über die Herausforderungen hinausgehen, die sich im Hinblick auf pathogene Mikroorganismen stellen und an anderer Stelle bewertet worden sind [4]. Ausgehend von den im geklärten Abwasser vorhandenen Kontaminanten und resistenten Mikroorganismen betrachten wir zunächst deren Effekt auf die bewässerten Pflanzen. Anschließend analysieren wir den Effekt der Verarbeitung der Lebensmittel auf die Konzentrationen von Antibiotikarückständen und Bakterien im verzehrfertigen Lebensmittel.

## Resistente Bakterien, Plasmide und Antibiotikarückstände im Zu- und Ablauf von Klärwerken

### Vorkommen resistenter Bakterien

Das Vorhandensein resistenter Mikroorganismen in aufbereiteten Abwässern ist weltweit gut belegt [6–10]. Resistente Mikroorganismen unterschiedlichster Art kommen in fast allen Abwässern vor [11]. Dabei unterscheiden sich Muster der feststellbaren Resistenzen zwischen Abwässern verschiedener Herkunft (kommunale Abwässer mit und ohne Krankenhaus im Einzugsgebiet, Schlachthofabwässer etc.; [8–10]). Die Zahl und Abundanz (Häufigkeit) der detektierten Resistenzgene ist auch von dem Behandlungsverfahren der Kläranlage abhängig [12].

Häufig ist unklar, in welcher Abundanz die resistenten Bakterien vorhanden sind. Bei kultivierungsunabhängigen DNA-basierten Nachweisverfahren ist meist nicht bekannt, ob es sich bei den festgestellten Genen um Nachweise aus intakten Bakterien oder aus freier DNA handelt, die nicht aus intakten Bakterien stammt, weil die Gene in direkt extrahierter DNA, d. h. ohne die vorherige Kultivierung und Isolierung der Bakterien, nachgewiesen werden. Insgesamt kann der Gehalt an Mikroorganismen stark variieren, je nach Jahreszeit und Temperatur sowie nach Aufbereitung der Abwässer (mechanisch, biologisch etc.) und Einspeisung aus verschiedenen Ressourcen [13].

Kultivierungsunabhängige DNA-basierte Untersuchungen zeigten, dass es im Laufe des Klärprozesses bei vielen Resistenzgenen zu einer Verminderung der Abundanz kommt [14]. Dabei bestehen sowohl hinsichtlich der Ausgangsabundanz [9] als auch hinsichtlich des Grades der Reduktion erhebliche Unterschiede [7, 15], die durch Verschiebungen in der Zusammensetzung der mikrobiellen Gemeinschaft im Rahmen des Klärprozesses begründet sind. Eine Übersicht über die nachgewiesenen Resistenzgene gaben Pazda et al. (2019; [11]).

Mit kultivierungsunabhängigen DNA- oder RNA-basierten Methoden können die Dynamik der Zusammensetzung des Mikrobioms sowie das Vorkommen von Antibiotikaresistenzgenen (ARG) oder mobilen genetischen Elementen (MGE) analysiert werden. Diese Methoden haben aber im Hinblick auf Empfindlichkeit und Informationen zum Resistenzphänotyp oder dem genetischen Kontext noch deutliche Schwächen gegenüber den traditionellen, oft mit Anreicherungen verbundenen Kultivierungstechniken. Letztere erlauben gleichzeitig die Charakterisierung des Resistenzphänotyps der isolierten Bakterien durch Gesamtgenomsequenzierung, die Analyse der plasmidlokalisierten Resistenzgene und ihrer Verknüpfung mit Insertionssequenz(IS)-Elementen [16] sowie den Vergleich von Isolaten aus aufbereitetem Abwasser und von rohverzehrtem Gemüse oder Obst. Die exogene Isolierung von übertragbaren plasmidlokalisierten Antibiotikaresistenzen in einem *E.-coli-* oder *Pseudomonas*-Rezipienten [17, 18] kann diese Analyse unterstützen.

### Rückstände antimikrobiell wirksamer Arzneimittel

Neben resistenten Mikroorganismen können geklärte Abwässer auch Rückstände antimikrobiell wirksamer Substanzen enthalten ([7, 13, 19–26]; exemplarisch Tab. 1). Auch bei den Rückständen kommt es im Rahmen der Klärung der Abwässer in der Regel zu einem Rückgang der Konzentrationen [23]. Es kann aber bei einigen Substanzen auch dazu kommen, dass durch chemische Prozesse die ursprüngliche Substanz aus Metaboliten wiederhergestellt wird, und deshalb die Konzentrationen im Auslauf von Kläranlagen höher sind als im Zulauf [22]. Die EU hat 5 antimikrobielle Substanzen auf eine Beobachtungsliste hinsichtlich der Belastung von Gewässern gesetzt: Ciprofloxacin, Amoxicillin, Erythromycin, Clarithromycin und Azithromycin [27]. Grundsätzlich ist davon auszugehen, dass es zukünftig durch verbesserte Technologien in der Abwasseraufbereitung zu einem Rückgang der im aufbereiteten Abwasser verbleibenden Rückstände kommen wird [5, 24, 28].

**Table 1:** 

Substanz	Minimum	Maximum	Anzahl Kläranlagen ohne Nachweis
Cefalexin	37	308	3
Trimethoprim	15,2	190,6	0
Ciprofloxacin	38,4	584,9	0
Enrofloxacin	69,4	69,4	12
Ofloxacin	20	305,1	1
Orbifloxacin	6,5	6,7	11
Clindamycin	6,5	110,7	1
Azithromycin	45,2	597,5	0
Clarithromycin	4,5	313,2	1
Metronidazol	7,6	93,2	4
Ampicillin	68,1	99,4	11
Nalidixinsäure	25,3	50,3	11
Oxolidinsäure	5,3	5,3	12
Pipemidsäure	3,2	30,1	1
Sulfamethoxazol	7,1	123,4	3
Sulfapyridin	4,7	184	0
Tetracyclin	15,4	231,2	2

In einer umfassenden Monitoringstudie zu aufbereitetem Abwasser von Kläranlagen in 7 EU-Mitgliedstaaten konnten 17 von 53 getesteten Antibiotika in mindestens einer Kläranlage nachgewiesen werden (Tab. 1). Im aufbereiteten Abwasser deutscher Kläranlagen wurden Trimethoprim, Ciprofloxacin, Ofloxacin, Azithromycin, Clarithromycin, Sulfamethoxazol, Sulfapyridin und Tetrazyklin nachgewiesen. Die Studie zeigte auch saisonale Unterschiede in der Antibiotikabelastung des aufbereiteten Abwassers. Von den Autoren dieser Studie wurden Ciprofloxacin, Azithromycin und Cefalexin als Marker für Antibiotikaverunreinigungen vorgeschlagen [13].

Diese Rückstände können einerseits zur weiteren Resistenzselektion im Mikrobiom der Abwässer beitragen. Andererseits können sie ggf. von den bewässerten Pflanzen aufgenommen werden [5] und dann als Rückstände im Lebens- oder Futtermittel eventuell toxikologisch relevante Wirkungen entfalten oder aber wiederum zur Resistenzselektion bei Pflanzen‑, Tier- und Menschen-assoziierten Mikroorganismen beitragen [29, 30].

Der Umfang, in dem die aufbereiteten Abwässer noch Wirkstoffe enthalten, hängt von der Ausgangssituation, also dem Eintrag der Wirkstoffe in die Kläranlagen, sowie dem Abbau oder der Sedimentation der Substanzen ab. Fraglich bleibt, inwieweit geringe Konzentrationen entsprechender Rückstände nach Ausbringung in die Umwelt zu einer Resistenzentwicklung führen. Subinhibitorische Rückstände unterbinden zwar nicht das Wachstum sensibler Organismen, könnten aber die Aufnahme von freier DNA mit Resistenzgenen begünstigen und damit einer weiteren Ausbildung von Resistenzen Vorschub leisten. Auch durch andere, nicht antimikrobielle Substanzen im Abwasser kann der Austausch mobiler genetischer Elemente befördert werden [31, 32]. Diese Veränderungen sind vor allem vor der Ausbringung zu erwarten, weil danach die Konzentrationen der Substanzen weiter sinken und die Verfügbarkeit der freien DNA in der Umgebung rasch abnimmt.

Während Beta-Laktam-Antibiotika in hohem Maße einem Abbau durch Hydrolyse unterliegen, besitzen andere Antibiotikaklassen wie Fluorchinolone, Tetrazykline, Makrolide und Sulfonamide eine höhere Stabilität. Sie werden nur langsam abgebaut und oft in der organischen Substanz des Klärschlamms abgelagert (sequestriert; [33]). Sie können sich ggf. in der Umwelt anreichern.

### Effekt zusätzlicher Behandlungsschritte im Klärprozess auf das Vorkommen von Mikroorganismen und Resistenzgenen

Weitere Aufbereitungsschritte des Abwassers, wie zum Beispiel die Ozonierung, können zu einer Reduktion der Zahl resistenter Bakterien und der Abundanz von Resistenzgenen beitragen. Sie führen aber aufgrund der unterschiedlichen Wirksamkeit gegenüber verschiedenen Mikroorganismen auch zu einer Verschiebung des Spektrums vorhandener Bakterien und ihrer Resistenzgene oder mobilen genetischen Elemente [7, 34]. Nur bei ausreichender Vorreinigung der Abwässer kann eine effiziente Reduktion von Mikroorganismen durch Ozonierung erreicht werden. Hierfür fehlen jedoch Daten, inwieweit die unterschiedlichen Prozessstufen einen wesentlichen Einfluss auf die mikrobielle Last der Abwässer ausüben. Daher empfehlen verschiedene Autoren eine Kombination von konventionellen Abwasserbehandlungsmethoden mit Verfahren, die auf oxidativen Methoden (UV-Strahlung, Ozon, H_2_O_2_) oder adsorptiven Methoden (Holzkohlefilter, Sandfilter und membranbasierten Filtertechnologien) basieren [5, 7, 24, 28].

## Kontamination von Pflanzen bei der Bewässerung mit aufbereiteten Abwässern

### Das Pflanzenmikrobiom

Die Zusammensetzung des Pflanzenmikrobioms (Gesamtheit aller mit Pflanzen assoziierten Mikroorganismen) hängt von der Pflanzenart, vom Wachstumsstadium, vom Mikrohabitat (Blatt, Stängel, Wurzel, endophytisch oder epiphytisch), dem Substrat, dem landwirtschaftlichen Management (organische Dünger, Bewässerung) und der Witterung ab. Von besonderer Bedeutung ist das Mikrobiom von roh verzehrten Pflanzen, wie Blattsalaten, Lauch und Wurzelgemüsen, da ihr Verzehr eine direkte Beeinflussung des menschlichen Darmmikrobioms ermöglicht [35]. Grundsätzlich dominieren bei den Bakterien die Proteobakterien und bei den Pilzen Basidiomyceten im Pflanzenmikrobiom [36]. Gerade Proteobakterien sind als genetischer Ursprung für eine Vielzahl mobiler Resistenzgene identifiziert worden, die eine klinische Relevanz aufweisen [37]. Der Einfluss des Bodentyps auf das Mikrobiom der Pflanzen wurde in verschiedenen Studien nachgewiesen [38]. Neben der Diversität werden auch das Vorkommen und die Persistenz von antibiotikaresistenten Bakterien durch die Beschaffenheit des Bodens beeinflusst [38, 39].

Epiphytische und auch die endophytischen Bakterien haben eine sehr heterogene Verteilung mit lokal sehr hohen Zelldichten (sogenannte Mikroaggregate, Biofilme), die die heterogene Verteilung und Verfügbarkeit von Nährstoffen für Mikroorganismen widerspiegelt. Die Phyllosphäre besiedelnde Bakterien haben häufig sehr effiziente Effluxpumpen, sie bilden Auxine, Antibiotika, Pigmente, Biosurfactants (Detergenzien), N‑Acyl-Homoserinlactone oder extrapolymere Substanzen, um sich an das Leben in der Phyllosphäre anzupassen [40, 41]. Dabei dienen die mikrobiell gebildeten Antibiotika als hochaktive Signalmoleküle. Es ist deshalb nicht überraschend, dass ein großer Anteil der pflanzenassoziierten Bakterien Mehrfachresistenzen gegen klinisch relevante Antibiotika besitzt. Die Kenntnisse zur Gesamtheit der natürlich vorhandenen Resistenzgene im Pflanzenmikrobiom (intrinsisches Resistom) wurden durch Metagenomstudien wesentlich verbessert [41–43].

Viele der typischen pflanzenbesiedelnden Gattungen (*Acinetobacter, Burkholderia, Enterobacter, Pantoea, Klebsiella, Pseudomonas, Stenotrophomonas* oder *Serratia*) spielen auch im Krankenhaus als Erreger nosokomialer Infektionen eine große Rolle. Der Erfolg von Isolaten dieser Gattungen im Habitat Krankenhaus erklärt sich durch effiziente Effluxpumpen und die Fähigkeit, Biofilme zu bilden. Dadurch sind sie bestens geeignet trotz des starken Selektionsdrucks im Krankenhaus (hoher Einsatz von antimikrobiellen Substanzen und Bioziden) aufgrund fehlender Konkurrenz zu kolonisieren.

Es ist wichtig festzuhalten, dass das natürliche Pflanzenmikrobiom nicht nur von großer Bedeutung für die Gesundheit und das Wachstum der Pflanzen ist, sondern auch für die Gesundheit des Menschen. Die Produktion von möglichst keimarmem Gemüse ist daher nicht sinnvoll. Vielmehr trägt eine hohe Diversität des Pflanzenmikrobioms dazu bei, dass eingebrachte Mikroorganismen, z. B. Keime aus dem Bewässerungswasser, sich nicht auf Pflanzen etablieren können. Pro Gramm Blattmaterial im Freiland gewachsenem Salat können typischerweise 10^6^–10^7^ koloniebildende Einheiten (KbE) nach einer Plattierung auf nichtselektiven Nährmedien nachgewiesen werden [44]. Diese Zahl wird nur in geringem Maße durch Waschen reduziert [45–47]. Man geht heute davon aus, dass das Samenmikrobiom und der Boden die Zusammensetzung des oberirdischen Pflanzenmikrobioms wesentlich beeinflussen [48]. Allerdings tragen auch eine Reihe von externen Faktoren wie Regen- und Bewässerungswasser, Staub- oder Boden-assoziierte Mikroorganismen zum Blatt-Mikrobiom bei.

### Einfluss der Bewässerung auf Mikrobiom und Resistom

Trotz der Belastung von aufbereitetem Abwasser mit antibiotikaresistenten Bakterien hat ihr begrenzter Nachweis im Boden zu der Einschätzung geführt, dass Boden wie ein Puffer wirkt, d. h. biotische und abiotische Bodenfaktoren den Effekt limitieren [49].

Bei der vergleichenden Nutzung von Oberflächen- und Trinkwasser oder aufbereitetem Abwasser zur Bewässerung zeigte sich in einer Metagenomstudie keine Beeinflussung des Bodenmikrobioms und -resistoms [50]. Wurde der Boden aber bei 37 °C in flüssigem Nährmedium (Lysogeny Broth) angereichert, zeigten sich deutliche Unterschiede. So wurden aus Böden, die mit aufbereitetem Abwasser beregnet worden waren, vor allem *Moraxellaceae* und *Enterobacteriaceae* angereichert, während aus Leitungswasser-beregneten Böden vor allem *Bacillaceae* angereichert wurden. Auch unterschieden sich 50 % der analysierten Resistenzgene signifikant. In dem Boden, der mit aufbereitetem Abwasser beregnet worden war, waren nach Anreicherung mehr als 61 % Multidrug-Resistance-Effluxpumpen (Membrantransporter, die antimikrobielle Stoffe aus Bakterien ausschleusen können) nachweisbar, im Leitungswasser-beregneten lediglich 8 %. In der Risikobeurteilung sollte berücksichtigt werden, dass bei Persistenz einer geringen Anzahl von Bakterien im aufbereiteten Abwasser unter geeigneten Bedingungen wieder eine Vermehrung stattfinden kann.

Aus Bewässerungswasser stammende Keime mit Mehrfachresistenz-Plasmiden können Pflanzen kolonisieren und auch Plasmide übertragen [51, 52]. Die Kombination aus Keimen mit übertragbaren, plasmidlokalisierten Antibiotikaresistenzen und Schadstoffen wie Antibiotika und Bioziden kann Effekte haben, die über die Einzeleffekte der mikrobiellen Kontamination und der Arzneimittelrückstände hinausgehen, und ist deshalb gesondert zu berücksichtigen. Auch wenn die Keimbelastung in aufbereitetem Abwasser im Vergleich zum ungereinigten geringer ist und z. B. *E.-coli*-Keimzahlen unter den zugelassenen Grenzwerten liegen, können sich solche Keime auf Pflanzen etablieren, insbesondere wenn sie Plasmide enthalten. Für einige typischerweise in Enterobakterien vorkommende Plasmide, wie die der IncF-Gruppe, konnte gezeigt werden, dass sie zur Ausbildung von Biofilmen beitragen können. Die im aufbereiteten Abwasser enthaltenen und von Pflanzen zum Teil aufgenommenen Arzneimittelrückstände und Biozide bieten den aus dem Bewässerungswasser stammenden Keimen einen Selektionsvorteil [53, 54]. Aufgrund der hohen Zelldichten in Mikrokolonien kann es darüber hinaus zur Übertragung von Plasmiden aus Bewässerungswasser-assoziierten Bakterien auf dominante Blatt-assoziierte Bakterien, wie z. B. die Enterobacterales und Pseudomonadales, kommen.

Antibiotika aus zur Bewässerung genutztem aufbereiteten Abwasser können in Abhängigkeit von ihren physikochemischen Eigenschaften von Pflanzen aufgenommen werden [25, 55]. Die Aufnahme und Akkumulation von Pharmazeutika und Pflegeprodukten aus dem Bewässerungswasser in Pflanzen sind auch von der Pflanzenart abhängig [30, 53, 54, 56–60]. Insbesondere in Gurken wurden erhöhte Konzentrationen gefunden [59, 61]. In den Pflanzen können sie das intrinsische Resistom modulieren, horizontale Gentransferprozesse stimulieren und die Etablierung von Abwasserbakterien auf Pflanzen fördern.

Die Charakterisierung von Tetrazyklin- und Colistin-resistenten und ESBL-produzierenden Enterobakterien aus Anreicherungen von Salat, der hydroponisch in verschieden aufbereitetem Abwasser gewachsen war, zeigte, dass sie zu den Gattungen *Enterobacter, Citrobacter, Klebsiella* und *Escherichia *gehören [62]. Die intrinsisch auf den Chromosomen vorkommenden AmpC-Beta-Laktamasen wurden in Isolaten des *Enterobacter*-Komplexes und von *Citrobacte*r spp. nachgewiesen. Als mobile, nicht chromosomal gebundene genetische Elemente gehörten die am häufigsten in Isolaten nachgewiesenen Plasmide zu den Inkompatibilitätsgruppen ColE1 und IncF. Aus Anreicherungen von aufbereitetem Abwasser und Salatblättern konnten konjugative Tetrazyklin-Resistenzplasmide in *E.-coli*-Rezipienten isoliert werden, die zu den Plasmiden der IncF-, IncI-, IncN- und der IncP-1-Gruppen gehörten. In direkt extrahierter DNA konnten IncF- und IncI-Plasmide nicht nachgewiesen werden, wohl aber in der nach Anreicherung extrahierten DNA [62].

### Entwicklung einer resistenten Mikroflora nach der Ernte durch Waschen oder andere Prozessschritte

Vor allem pflanzliche Produkte, die zum Rohverzehr geeignet sind, stellen eine sensible Produktgruppe für eine potenzielle Übertragung von antibiotikaresistenten Bakterien auf den Menschen dar [63]. Bei diesen Produkten (Obst, Gemüse) fehlt oftmals ein Dekontaminationsschritt wie das Erhitzen. Eine Verarbeitung wie Waschen oder Schälen ermöglicht nur eine moderate Reduktion von Mikroorganismen. Daher werden diese Produkte im Folgenden fokussiert betrachtet. Insbesondere für vulnerable Gruppen ist es dabei wichtig, die Hinweise über den sicheren Umgang mit solchen Lebensmitteln zu beachten [64].

Bei der Betrachtung von Antibiotikaresistenzen in pflanzlichen Lebensmitteln nehmen vor allem Enterobakterien eine hervorgehobene Stellung ein. Enterobakterien wie *E. coli, Klebsiella*, aber auch *Enterobacter* und *Citrobacter* gehören zu den häufigsten Infektionserregern und sind aufgrund von Antibiotikaresistenzen oftmals schwer zu therapieren [65]. Gleichzeitig sind Pflanzen natürlicherweise mit Enterobakterien besiedelt [66]. Der Anteil der Enterobakterien am gesamten Mikrobiom beträgt meist nur 1–5 %, ist aber von einer hohen Varianz gekennzeichnet [66]. Cho et al. konnten antibiotikaresistente *Enterobacter*-, *Citrobacter*-, *Escherichia*- und *Klebsiella*-Arten aus pflanzlichen Frischeprodukten des deutschen Einzelhandels isolieren [67]. Mittels Genomsequenzierung wurden Resistenzgene gegen Tetrazykline, Sulfonamide, Aminoglykoside, Chloramphenicol, Chinolone und Beta-Laktamantibiotika nachgewiesen. Des Weiteren zeigten die Isolate verschiedene Plasmidgruppen wie IncF, IncH, IncN, IncQ, IncR, p0111 und ColRNAI, was auf eine Mobilisierung der Resistenzgene hindeutet [67]. Blau et al. untersuchten das Vorkommen von Plasmiden (IncF, IncI, IncP‑1 und Klasse 1 Integrons) auf kommerziell erworbenem Koriander, Rucola und Mischsalat [17]. Insbesondere die IncF- und IncI-Plasmide in der Gesamt-DNA konnten nur nach vorheriger Anreicherung detektiert werden. Gekenides et al. (2018) zeigten, dass Bewässerungswasser eine wichtige Quelle von antibiotikaresistenten Bakterien auf Frischeprodukten ist [68].

Pflanzen- und produktgruppenspezifische Unterschiede führen zu verschiedenen mikrobiologischen Belastungen in Obst und Gemüse. Üblich sind Keimgehalte von ca. 4–8 log KbE/g in der Gesamtkeimzahl und 3–7 log KbE/g Enterobakterien [44]. Sprossen sind beispielsweise höher belastet als Salatgurken [66]. Generell sind bodennah wachsende Pflanzenteile höher belastet. Die Keimzahlen von Pflanzen aus dem Gewächshaus sind 2–3 log KbE/g niedriger als Keimzahlen von Pflanzen aus dem Freiland.

Bei frischen pflanzlichen Lebensmitteln, wie Obst und Gemüse, bleibt der Stoffwechsel auch nach der Ernte aktiv. Neben der Respiration (Aufnahme von Sauerstoff, Abgabe von Kohlenstoffdioxid) führen vor allem intrazelluläre Abbaureaktionen zu einer Veränderung der physikalisch-chemischen Beschaffenheit dieser Lebensmittel. Bakterien, Hefen und Schimmelpilze unterstützen diese Prozesse und führen zum mikrobiologischen Verderb.

Meist ist Gemüse und Obst durch eine intakte Oberfläche geschützt. Diese verhindert den Austritt von nährstoffreichem Zellsaft und schützt vor einer raschen Vermehrung von Mikroorganismen (insbesondere schnell wachsende Bakterien wie *E. coli*). Allerdings gibt es Produktgruppen, wie vorgeschnittenes Obst oder geschnittene und gewaschene Salate („Fresh-Cut“), deren zerstörte Oberflächenstruktur eine rasche Vermehrung von Mikroorganismen zulässt. Um das Wachstum zu verlangsamen, müssen geschnittene Produkte gekühlt werden. Dies ist grundsätzlich bei intakter Oberfläche nicht notwendig, da sich Mikroorganismen während der üblichen Lagerzeit nicht zu einem beeinträchtigenden Maße vermehren.

Die Verarbeitungsschritte, wie Waschen und Schneiden, beeinflussen die Mikrobiota der Produkte deutlich. Das industrielle Waschen von pflanzlichen Produkten führt zu einer gewissen Reduktion der mikrobiellen Belastung, aber auch zu einer Verteilung der Mikroorganismen zwischen den gewaschenen Produkten [47, 66]. Die Übertragung von pathogenen und antibiotikaresistenten Bakterien kann dadurch auch zwischen den Produkten stattfinden. Im Fresh-Cut-Bereich wurde durch den Waschschritt eine moderate Reduktion von ca. 0,27 log KbE/g in der Gesamtkeimzahl und ca. 0,36 log KbE/g bei der Enterobakterienanzahl festgestellt [66]. Mit einer Nachweisrate von 10 % (*L. monocytogenes*, Shigatoxin-bildende *E. coli, Salmonella* spp.) wies die Produktgruppe der Fresh-Cut-Salate eine deutlich höhere Belastung mit humanpathogenen Bakterien auf als frische Kräuter, Kopf‑/Blatt‑/Pflücksalate, Sprossen, Möhren, Speisepilze und Gurken [66]. Daher ist durch den Waschschritt auch mit einer Verteilung von antibiotikaresistenten Bakterien zu rechnen.

Die Reduktion der mikrobiellen Besiedlung durch die gesamte Prozessierung von Fresh-Cut-Salaten (Vorputzen, Schneiden, Waschen) beträgt etwa 0,5 log KbE/g in der Gesamtkeimzahl und etwa 1 log KbE/g in der Anzahl der Enterobakterien [66]. Als marktfähige Einheit kommt es während der Haltbarkeitsdauer zu einem Anstieg der Keimzahlen um ca. 1 log KbE/g in der Gesamtkeimzahl und ca. 2 log KbE/g bei der Enterobakterienanzahl [69].

Durch das Waschen im Privathaushalt wurden lediglich geringfügige Reduktionen von 0,5–0,9 log KbE/g in der Gesamtkeimzahl und 0,3–0,7 log KbE/g bei den Enterobakterienzahlen beschrieben [45, 46]. Der Waschvorgang bei Gemüse und Salat eignet sich demnach grundsätzlich nicht, um eine mikrobiologische Sicherheit zu gewährleisten, sondern nur um ein potenzielles Risiko geringfügig zu mindern.

### Lagerung

Deutschland ist ein Importland für Gemüse und Obst (64–78 % werden importiert). Auch wenn der Großteil in anderen EU-Ländern (Niederlande/Spanien) produziert wird, findet ein globalisierter Handel mit pflanzlichen Frischeprodukten statt. Durch Transport und Vorratslagerung können Lagerzeiten von mehreren Wochen entstehen. Trotz verbesserter Lagerungsbedingungen kommt es zu Veränderungen in der mikrobiologischen Besiedlung. Bei Kühllagerung kann es beispielsweise zu einer Vermehrung von kältetoleranten Enterobakterien (z. B. Yersinien; [69]) oder anderen Veränderungen des Mikrobioms und Resistoms kommen [42].

Durch das Verpacken in Kunststoffverpackungen mit modifizierter Atmosphäre wird das Wachstum von Mikroorganismen gehemmt [70]. Im Fresh-Cut-Bereich werden verschiedene Systeme eingesetzt. *Modified Atmosphere Packages* (MAP) besitzen eine produktspezifisch modifizierte Schutzatmosphäre von 3–8 % CO_2_, 2–5 % O_2_, 87–95 % N [71]. Ein weiteres System ist das *Equilibrium Modified Atmosphere Packaging *(EMA), indem die Verpackung eine definierte Permeabilität aufweist und der Gasaustausch zu einem Equilibrium zwischen Austausch und Respiration der Pflanzen/Mikroorganismen führt [71]. Sowohl MAP als auch EMA führen zu einem reduzierten Sauerstoffgehalt in der Verpackung, wodurch aerobe Mikroorganismen gehemmt und anaerobe Bakterien begünstigt werden [70]. Enterobakterien können sich als fakultativ anaerobe Bakterien auch unter diesen Bedingungen vermehren und ihren Anteil an der Mikrobiota erhöhen [72].

## Fazit

Pflanzen sind grundsätzlich kein natürliches Habitat für fäkale Enterobakterien wie *E. coli*. Jedoch bilden Pflanzen ein allgemeines Reservoir für Enterobakterien, was auch medizinisch relevante Gattungen wie *Escherichia, Klebsiella, Enterobacter* und *Citrobacter* einschließt [73]. Prinzipiell können diese Bakterien auch Antibiotikaresistenzen tragen und weitergeben [65]. Der Eintrag von antibiotikaresistenten Bakterien kann über Düngung, Wild- und Haustiere, Luft, Wasser sowie menschliche Einflüsse auf Böden und auf die Pflanzen stattfinden [74]. Ein Eintrag über Bewässerungswasser findet statt, wird aber nicht als dominierende Eintragsquelle beschrieben. Er folgt denselben Prinzipien wie der Eintrag von humanpathogenen Bakterien. Die in der EU-Verordnung 2020/741 definierten Anforderungen an die Wasserqualität von Bewässerungswasser reduzieren die Wahrscheinlichkeiten für eine Kontamination der Pflanzen mit antibiotikaresistenten Bakterien analog zu pathogenen Bakterien. Der Indikatorkeim *E. coli* wird als genereller Parameter für das Vorkommen von fäkalen Enterobakterien verwendet. Seine Abwesenheit oder eine geringe Anzahl in Bewässerungswasser lässt sich demnach als Qualitätsparameter auch auf andere, potenziell antibiotikaresistente Enterobakterien übertragen.

Spezielle Verarbeitungs- und Lagerbedingungen wie im Fresh-Cut-Bereich können eine Vermehrung von Enterobakterien begünstigen. Die Eigenschaft der Antibiotikaresistenz führt nicht grundsätzlich zu einem veränderten Wachstumsverhalten der Mikroorganismen, einschließlich der Humanpathogene. Dadurch ist die im Hinblick auf pathogene Bakterien getroffene Einschätzung zu den mikrobiellen Anforderungen an das Bewässerungswasser auch auf antibiotikaresistente Bakterien übertragbar.

Einer besonderen Betrachtung bedarf allerdings die Exposition gegenüber einer Kombination von Mikroorganismen mit übertragbaren Resistenzeigenschaften und Spuren von Antibiotikarückständen oder Mikroschadstoffen im Bewässerungswasser. Diese Kombination kann zu einer Begünstigung des Wachstums der resistenten Mikroorganismen auf den bewässerten Pflanzen und zu einer Weitergabe der mobilen genetischen Elemente führen. Bei den Mikroorganismen im Bewässerungswasser wie auch bei Klärschlamm handelt es sich um potenziell humanadaptierte Stämme und bei den Rückständen um Substanzen, die zur Therapie von bakteriellen Infektionen beim Menschen verwendet werden. Die Exposition erfolgt nicht einmalig, wie bei Düngemitteln, sondern über einen bestimmten Zeitraum mehr oder weniger kontinuierlich. Die durch diese Kombinationen induzierten Veränderungen und Interaktionen der Mikrobiota auf Pflanzen sind wenig erforscht, sodass weitere Studien im Hinblick auf Antibiotikaresistenzen und die Persistenz der von Pflanzen aufgenommenen Mikroschadstoffe notwendig erscheinen.

Aktuell gibt es vermehrt Hinweise, dass neben antibiotischen Substanzen auch andere Spurenstoffe den Gentransfer und somit auch einen Resistenzgenaustausch beschleunigen können, z. B. künstliche Süßstoffe [31, 32, 75].

Unter diesem Gesichtspunkt ist neben den mikrobiologischen Anforderungen auch die Minimierung des Gehaltes an antimikrobiellen Substanzen zu fordern. Als Orientierung kann dabei die im Durchführungsbeschluss (EU) 2018/840 vorgeschlagene Liste von antimikrobiellen Substanzen berücksichtigt werden.
